# Oxidative Stress in Disease and Aging: Mechanisms and Therapies 2016

**DOI:** 10.1155/2017/4310469

**Published:** 2017-01-26

**Authors:** Claudio Cabello-Verrugio, Felipe Simon, Capucine Trollet, Juan F. Santibañez

**Affiliations:** ^1^Laboratorio de Biología y Fisiopatología Molecular, Departamento de Ciencias Biológicas, Facultad de Ciencias Biológicas and Facultad de Medicina, Universidad Andres Bello, Santiago, Chile; ^2^Millennium Institute on Immunology and Immunotherapy, Santiago, Chile; ^3^Laboratorio de Fisiología Integrativa, Departamento de Ciencias Biológicas, Facultad de Ciencias Biológicas and Facultad de Medicina, Universidad Andres Bello, Santiago, Chile; ^4^Sorbonne Universités, UPMC Univ Paris 06, Centre de Recherche en Myologie, INSERM UMRS974, CNRS FRE3617, Institut de Myologie, 47 bd de l'Hôpital, 75013 Paris, France; ^5^Laboratory for Experimental Hematology and Stem Cells, Institute for Medical Research, University of Belgrade, Serbia; ^6^Laboratorio de Bionanotecnologia, Universidad Bernardo O'Higgins, Santiago, Chile

Oxidative stress (OS) is an imbalance between the formation of reactive oxygen species (ROS) and antioxidant defense mechanisms. This phenomenon increases with age and affects the normal functioning of several tissues. Furthermore, numerous chronic diseases associated with older age, such as diabetes and cardiovascular, renal, pulmonary, and skeletal muscle disorders, are also directly related to OS. Considering this relationship, the aim of many ongoing studies is to elucidate the underlying mechanisms and role of OS in disease onset and development. In particular, there is considerable emphasis on finding new therapeutic strategies for decreasing OS.

This special issue presents new and relevant research regarding the mechanisms by which OS induces oxidative damage in the contexts of chronic disease and aging. Focus is given to the use of novel antioxidant strategies. The manuscripts within this special issue are all equally recommended by the editors, but the following contain especially interesting points worth comment.

X. H. Yang et al. evaluated the effect of epigallocatechin-3-gallate (EGCG), a component derived from green tea, on OS in the kidney of diabetic db/db mice. They demonstrated that EGCG ameliorated the levels of several parameters associated with OS and of signaling pathways related to oxidative damage and renal fibrosis (e.g., RAS axis, NOX, MAPK, TGF-b, and a-SMA). Overall, a renoprotector effect was found in diabetic mice.

N. Mihailović-Stanojević et al. studied the effect of wild thyme, a spice plant rich in polyphenolic compounds, on the development of hypertension. The authors demonstrated that wild thyme decreased OS and blood pressure in hypertensive rats, probably by increasing heme oxygenase 1.

E.-M. Noh et al. investigated how ROS production in senescent fibroblasts is generated by the modulation of phosphatidylinositol 3,4,5-triphosphate metabolism. The study showed in replicative senescent cells that increased ROS production was blocked by inhibiting three key signaling pathways: PI3K, protein kinase C, and NADPH oxidase. Additionally, the results indicated that a reduction in PTEN levels is important for abolishing increased ROS levels in human dermal fibroblasts.

J. Abrigo et al. studied the effect of mesenchymal stem cell administration on skeletal muscle damage induced by a high fat diet. The authors demonstrated that muscle wasting was produced by activating the ubiquitin proteasome pathway increased OS levels and myonuclear apoptosis. The main finding was that mesenchymal stem cells abolished these three mechanisms involved in the muscle damage induced by a high fat diet.

All of these highlighted studies, as well as the other manuscripts contained in this special issue, advance improvements on pathological statuses by using diverse antioxidant strategies. We firmly believe that these findings will be of relevance to research concerning OS, chronic diseases, and aging.

